# A computational model for preplay in the hippocampus

**DOI:** 10.3389/fncom.2013.00161

**Published:** 2013-11-12

**Authors:** Amir H. Azizi, Laurenz Wiskott, Sen Cheng

**Affiliations:** ^1^Mercator Research Group “Structure of Memory,” Department of Psychology, Ruhr-University BochumBochum, Germany; ^2^Institute for Neural Computation, Ruhr-University BochumBochum, Germany

**Keywords:** continuous attractor neural networks, multi-chart structure, spike-frequency-adaptation, sequential activity, preplay

## Abstract

The hippocampal network produces sequences of neural activity even when there is no time-varying external drive. In offline states, the temporal sequence in which place cells fire spikes correlates with the sequence of their place fields. Recent experiments found this correlation even between offline sequential activity (OSA) recorded before the animal ran in a novel environment and the place fields in that environment. This preplay phenomenon suggests that OSA is generated intrinsically in the hippocampal network, and not established by external sensory inputs. Previous studies showed that continuous attractor networks with asymmetric patterns of connectivity, or with slow, local negative feedback, can generate sequential activity. This mechanism could account for preplay if the network only represented a single spatial map, or chart. However, global remapping in the hippocampus implies that multiple charts are represented simultaneously in the hippocampal network and it remains unknown whether the network with multiple charts can account for preplay. Here we show that it can. Driven with random inputs, the model generates sequences in every chart. Place fields in a given chart and OSA generated by the network are highly correlated. We also find significant correlations, albeit less frequently, even when the OSA is correlated with a new chart in which place fields are randomly scattered. These correlations arise from random correlations between the orderings of place fields in the new chart and those in a pre-existing chart. Our results suggest two different accounts for preplay. Either an existing chart is re-used to represent a novel environment or a new chart is formed.

## 1. Introduction

While we know since patient HM that the human hippocampus is involved in the formation and consoldation of episodic memories (Scoville and Milner, 1957), the neural mechanisms underlying these processes are still not understood. One promising candidate for a mechanism underlying consolidation is the reactivation of neural activity patterns during sleep and awake quiescent states (offline states) (McClelland et al., 1995). During offline states, hippocampal place cells fire spikes in a temporal order that correlates with the order of place fields recorded during earlier exploration (replay) (Lee and Wilson, 2002; Foster and Wilson, 2006; Buhry et al., 2011). The neural mechanism underlying replay were thought to be relatively simple. External sensory inputs during exploration are thought to drive plasticity and imprint the sequence of neural activity, e.g., by an asymmetric plasticity rule along with the theta phase precession (Wagatsuma and Yamaguchi, 2007). In the offline state, the imprinted sequences are evoked by noisy spiking events, perhaps driven by the unstructured and non-sensory inputs observed in the hippocampus in this state (Buzsáki, 1996). Subsequent experiments confirmed the functional relevance of replay activity in memory acquisition and consolidation. Since replay events are accompanied by sharp wave/ripple (SWR) events in the hippocampal local field potential (LFP), it is possible to detect SWR and electrically stimulate the hippocampus to suppress replay activity. When this protocol was applied during sleep (Girardeau et al., 2009) or the awake state (Jadhav et al., 2012), rats showed significant impairments on spatial memory tasks.

However, this view of sequential activity in the hippocampus does not readily account for recent observations (Buhry et al., 2011): (1) Place cells that have fields in a novel environment are reactivated more frequently and fire more spikes than cells representing familiar environments (Cheng and Frank, 2008), suggesting that the frequency of reactivation does not match the amount of sensory experience. (2) In a significant number of cases, offline sequential activity (OSA) represents trajectories in space that were never experienced by the animal (Gupta et al., 2010). (3) The order of OSA recorded before the animal experiences a novel environment is predictive of the order of place fields that emerged in that environment (Dragoi and Tonegawa, 2011). (4) Some spatial trajectories represented by OSA were actually traversed during later exploration by the animal to get to a goal location that was unknown at the time of the OSA (Pfeiffer and Foster, 2013). In summary, the types of OSA reviewed above cannot be the result of a sensory-driven learning mechanism that imprints sequences into the hippocampal network. Instead, these observations suggest that OSA stem from the intrinsic structure of the network. We recently proposed a conceptual framework for the hippocampal formation, in which the massive recurrent connections in region CA3 generate intrinsic sequences, which are used to represent episodic memories (Cheng, 2013). Here we propose a computational model for how CA3 might generate these intrinsic sequences to account for preplay.

We base our model of area CA3 on continuous attractor neural network (CANN), which have been employed previously to generate sequential activity. In a CANN, local excitation and global inhibition are balanced to allow for a stable network state, i.e., a localized bump of activity. Its position can be perturbed by introducing asymmetric synaptic connections (Tsodyks et al., 1996; Tsodyks, 1999; Burak and Fiete, 2009), asymmetric inputs (Ben-Yishai et al., 1995; Samsonovich and McNaughton, 1997; McNaughton et al., 2006), or slow and local negative feedback (Wiskott and von der Malsburg, 1996; Pinto and Ermentrout, 2001; Richardson et al., 2005; Itskov et al., 2011b). A related network was used to model mental exploration across different environments (Hopfield, 2010). Therefore, if the hippocampal network only represented a single spatial map, then a CANN with a moving bump could account for preplay. The picture, however, is complicated by the fact that hippocampal place cells switch their spatial representation when the animal moves between sufficiently distinct environments (Muller and Stead, 1996). This global remapping effect implies that multiple mappings from hippocampal neurons to physical space, charts, must co-exist in the network (Samsonovich and McNaughton, 1997). Recently, Dragoi and Tonegawa (2013) found that disjoint sets of pre-run OSAs are correlated with the order of place fields in different novel tracks. Based on this observation they suggest that only a relatively small number of charts are encoded in the hippocampal network. While models of path integration with multiple context representations have been discussed before (Colgin et al., 2010), the models cannot account for sequential reactivation observed during the offline states. In principle, any model that produces sequences intrinsically and in which cells are assigned place fields *post-hoc* in novel environments might be able to account for preplay. However, to the best of our knowledge, no study has shown quantitatively that the activity generated by a biologically plausible model accounts for the preplay phenomenon. Here we base our modeling on the continuous attractor neural network (CANN) with spike-frequency adaptation coupled with the multiple chart idea (Hopfield, 2010) to show that this network can generate OSA that is correlated with the order of place fields in a novel environment. This correlation arises intrinsically in the network, and there is no need for idiothetic information or *a priori* learning mechanisms.

## 2. Materials and methods

### 2.1 Network dynamics

**Figure 1:**
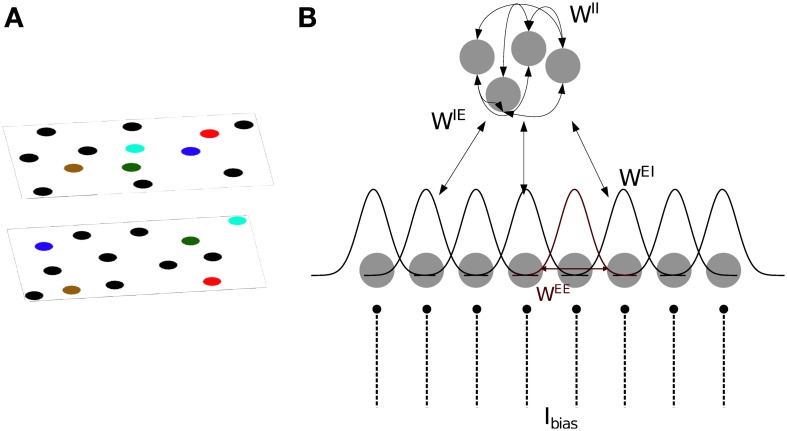
**Model definition. (A)** The two dimensional arrangement of excitatory cells represents a map of the environment, so that neighboring cells have neighbouring place field centers (PFC). By randomly rearranging the PFC's of the cells, the same neurons can represent different environments (multiple charts). **(B)** One dimensional representation of the network architecture. 80% of the units in the network are excitatory (lower layer) and the remaining 20% are inhibitory neurons (top layer). There are all-to-all connections between excitatory and inhibitory neurons and among inhibitory neurons, denoted by *W*^IE^, *W*^EI^ and *W*^II^. As a result, each excitatory neuron receives global inhibition that is a function of the total network activity. Each excitatory neuron also receives a constant bias input, *I*_bias_. The connectivity pattern between excitatory neurons in each chart, *W*^EE^, is a Gaussian function of the distance between their PFC's. The total excitatory weight matrix of the network is the summation of the weights in each chart.

We base our model of CA3 on two models proposed by Hopfield (2010) and Samsonovich and McNaughton (1997). The model consists of integrate- and fire neurons arranged in two sub-layers of excitatory and inhibitory units (Figure 1). The membrane potential of excitatory (*s* = E) and inhibitory (*s* = I) neurons is defined as follows:

(1) $$\frac{\text{d}u_{i}^{s}}{\text{d}t}=-\frac{u_{i}^{s}}{\tau_{\text{cell}}^{s}}+I_{\text{bias}}+I^{s\text{E}}-I^{s\text{I}}-J_{i}^{s}+I_{\text{noise}},$$

where *u*^*s*^_*i*_ is the membrane potential of the *i*-th neuron, and *I*_bias_ is a constant input current which determines the level of excitability of each neuron. In the absence of synaptic and noise inputs, the membrane potential relaxes to τ^*s*^_cell_*I*_bias_. τ^*s*^_cell_ = 20 ms is the integration time constant for both cell types. *I*_noise_ is a Gaussian noise input current with zero mean and standard deviation of 0.2.

Excitatory neurons receive inhibitory feedback of their activity through the adaptation current *J*^E^_*i*_. Inhibitory neurons receive no adaptation current *J*^I^_*i*_ = 0. This adaptation current models spike-frequency adaptation (SFA), i.e., the current causes the spiking frequency of the cell to decrease with each spike depending on the time elapsed since the spike,

(2) $$\frac{\text{d}J_{i}^{\text{E}}}{\text{d}t}=-\frac{J_{i}^{\text{E}}}{\tau_{\text{unadapt}}}.$$

Experimental data suggest that the time constant with which the adaptation current decays in CA3 pyramidal cells is very long compared to the cell integration time (Hemond et al., 2008). We use τ_unadapt_ = 5 s.

A spike is generated, when the membrane potential of a neuron reaches the firing threshold *u*_th_ = 1. In addition, the membrane potential is reset to *u*_reset_ = 0 and, if the neuron is excitatory, the adaptation current increased by α.

(3) $$\text{If }u_{i}^{s}(t)=u_{\text{th}}\text{ then }\left\{ \begin{array}{l} u_{i}^{s}\to u_{\text{reset}}^{s}\\ J_{i}^{s}\to J_{i}^{s}+\alpha \text{ if }s=\text{E.} \end{array}\right.$$

Each spike triggers a post-synaptic current given by

(4) $$\frac{\text{d}I^{ss^{\prime }}}{\text{d}t}=-\frac{I^{ss^{\prime }}}{\tau^{s^{\prime }}}+\sum_{j,k}W^{ss^{\prime }}(i,j)\delta \left(t-t_{jk}\right),$$

where *s*′ = {E,I} indicates the type of the sending neuron and *t*_*jk*_ is the *k*-th spike of the *j*-th neuron. The excitatory and inhibitory synaptic integration time constants are set to τ^E^ = 6 ms and τ^I^ = 4 ms, respectively. The weight matrices *W*^*ss*′^ determine the structure of the network and are described below.

### 2.2 The multi-chart structure

A chart is a mapping from neurons, represented by their integer index, to the 2-d space in which the animals move (Samsonovich and McNaughton, 1997).

(5) $$r:N\to R^{2}$$

Specifically, *r*_*i*_ represents the place field center (PFC) of neuron *i*. Multiple such mappings *r*^μ^_*i*_ can co-exist for a given set of neurons. The PFCs of the place cells are drawn independently from a uniform distribution across a box of size 1 × 1 m. In our network, each excitatory place cell is connected to its *M* nearest neighbors in a given chart. No autapses are allowed. The strength of the connections decreases with the distance between PFCs

(6) $$W_{\mu }^{\text{EE}}(i,j)=\frac{1}{\sqrt{2\pi }\sigma }\exp\left[-\frac{{\left(r_{i}^{\mu }-r_{j}^{\mu }\right)}^{2}}{2\sigma^{2}}\right].$$

The width of the kernel was chosen to be σ = 15 cm. The contribution of each chart to the total weight matrix is additive:

(7) $$W^{\text{EE}}(i,j)=\sum_{\mu =\;1}^{P}W_{\mu }^{\text{EE}}(i,j).$$

Bump attractors appear in the excitatory network only when the total network activity is limited by a combination of SFA and global inhibition. Every excitatory cell projects to all inhibitory cells through the random connections *W*^IE^, drawn from a uniform distribution between 0 and 0.05. Inhibitory cells are recurrently connected to one another via random weights *W*^II^, drawn from a uniform distribution between 0 and 0.17; and inhibit the excitatory cells through random connections *W*^EI^, drawn from a uniform distribution between 0 and 0.1 (Figure 1B). The range of these random weights are chosen such that the network generates a bump. The all-to-all connection to/from the inhibitory sub-layer ensures that each excitatory cell receives a global inhibitory feedback input current from the total activity of all the other excitatory cells.

### 2.3 Network simulations and bump analysis

The network consists of 2000 excitatory cells and 500 inhibitory cells. To sample from the distribution of networks, i.e., different placements of place field centers and the distribution of input noise, we perform Monte Carlo simulations with 20 network instantiations and *n* repetitions, which is specified for each result separately. The activity is initiated by providing *I*_bias_ = 1.92 to 400 excitatory cells, selected randomly, and to all the inhibitory cells. After the initiation period, *I*_bias_ = 1.92 is provided to all excitatory cells and *I*_bias_ = 1.62 to all the inhibitory cells. Euler integration was used with a time step of 0.5 ms to solve the differential equations.

Much of our analysis focuses on the dynamic properties of the bump attractor. To detect whether a bump was formed in a chart, we first determined which cells *i*_*k*_ were active in a 40 ms time window, then we computed the standard deviation of the network activity in the chart space (Samsonovich and McNaughton, 1997).

(8) $$\sigma_{\mu }^{2}=\frac{1}{N_{\text{active}}-1}\sum_{\text{k=1}}^{N_{\text{active}}}{\left(r_{i_{k}}^{\mu }-{\bar{r}}_{i_{k}}^{\mu }\right)}^{2}$$

for each chart. A bump was considered to exist in chart μ, if σ_μ_ < 30 cm.

### 2.4 Analysis of spatio-temporal correlations

To map a new environment, a subset of cells that were close together in a chart representation was selected. The activity of 20 cells *i*_*j*_ that were selected randomly out of this subset, was recorded during the simulation runs. The *x*-coordinate of the PFCs *x*_*i*_*j*__ of these cells were stored in the template vector. In this way, the template cells map a new environment maintaining the same metric relationship as in the chart that they were selected from.

Similar to Dragoi and Tonegawa (2013), we defined spiking events based on multi-unit activity. However, we did not require silent periods flanking the spiking event since we did not model oscillations such as sharp wave/ripples (SWR) that impose temporal structure on the spiking of neurons. Nonetheless, we assume that the OSA that our model generates occur during the SWR state, as shown before, such as in Dragoi and Tonegawa (2011, 2013). We used a sliding window of 100 ms width to identify spiking events in which at least 5 template cells fired action potentials. Once 5 of more cells were found to spike within the sliding window, it was adjusted to begin at the time of the first spike, which we defined as the beginning of the spiking event. The spiking event ended with the last spike within the sliding window. For each spiking event, we determined the time of the first spike *f*_*k*_ for the active template cells and calculated the rank-order correlation between *f*_*k*_ and *x*_*k*_. The rank-order correlation is defined as (Press et al., 1992, page 640):

(9) $$R=\frac{\sum_{k}\left(\xi_{k}-\bar{\xi })(\phi_{k}-\bar{\phi }\right)}{\sqrt{\sum_{k}{\left(\xi_{k}-\bar{\xi }\right)}^{2}}\sqrt{\sum_{k}{\left(\phi_{k}-\bar{\phi }\right)}^{2}}},$$

where ξ_*k*_ is the rank of neuron *k* in the list *x*_*k*_, and ϕ_*k*_ is its rank according to *f*_*k*_. The means of these values are represented by ξ and ϕ, respectively.

To assess statistical significance, the network-generated distributions of correlation values were compared to shuffled distributions. The latter were generated by correlating the *f*_*k*_'s with randomly permuted *x*_*k*_'s. The permutation was performed for each spiking event to ensure that any relationship between spike times and PFCs was destroyed in the shuffled distribution. We note that our shuffling procedure did not affect other variables such as the number of active cells per spiking event, which have a large influence on the shape of the distribution. To compare network-generated and shuffled distributions we used the two-sample Kolmogorov–Smirnov test with a cutoff of *p* = 0.01. Our results did not differ qualitatively when we used the Wilcoxon ranksum test on the absolute values of the correlations.

## 3. Results

### 3.1 Bump formation in a multi-chart continuous attractor network

**Figure 2:**
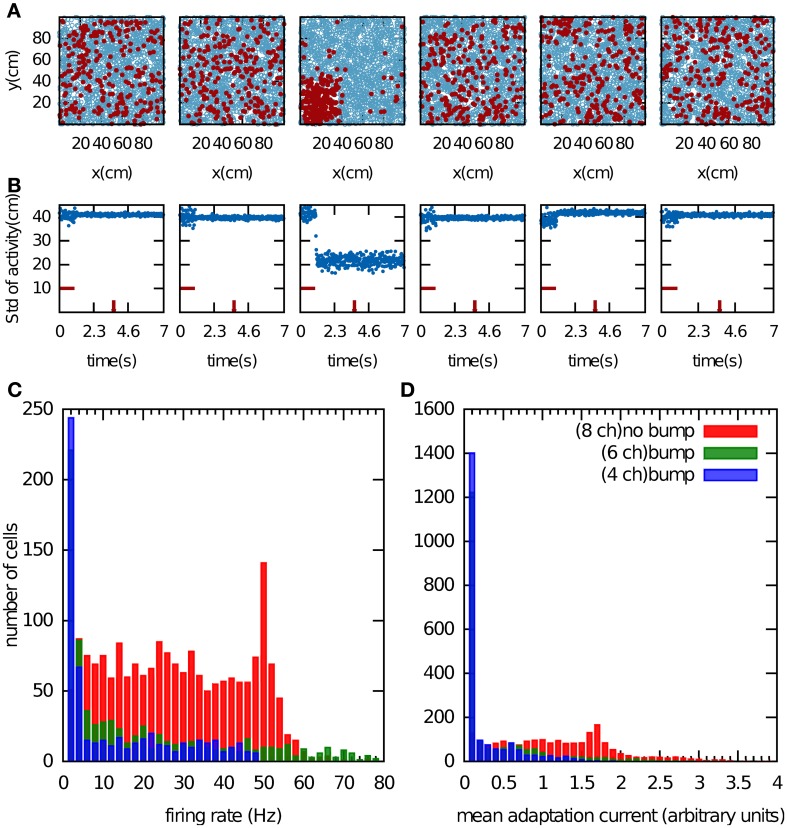
**Bump formation in the network without spike-frequency adaptation. (A)** Local excitation and global inhibition give rise to a bump attractor, a region of clustered activity in the excitatory layer. Because of the competitive nature of the network activity, the bump can form in only one chart (here the third chart). In the other charts, the activity appears randomly scattered. The place field centers are represented by blue circles. Spiking cell are indicted by the red filled circles. **(B)** Standard deviation of the activity as a function of time. The horizontal red bar indicates the time period during which a biased external input was applied to one fifth of the excitatory neurons, selected randomly. The time at which network activity is shown in **(A)** is indicated by the vertical red bar. **(C,D)**, Cell activity when the network stores 4 (blue bars), 6 (green bars) and 8 (red bars) charts. Shown are the distributions of the mean firing rate of the cells **(C)** and of the average adaptation input current, which was disconnected from the network dynamics **(D)**.

We first examined the properties of the bump attractor in a CANN that stores multiple charts with the adaptation currents *J*_*i*_ removed from the network dynamics in Equation 1. In this way, the adaptation current had no effect on the network dynamics but was allowed to accumulate. We return to this point in the next paragraph. The network activity was initialized by providing constant input currents for 1 s to one fifth of the population, which was selected randomly. After this period, the activity self-organized into a small region of the network (Figure 2A). This bump formed only in one chart. Since the charts were drawn independently of one another, the active cells were scattered seemingly randomly when arranged in a different chart (Figures 2A,B). Which chart the bump forms in, is determined by random heterogeneities in the network structure that break the symmetry. These heterogeneities are present in the distances between PFCs within one chart, in the cross-talk from other charts stored in the connectivity matrix, and the noise input.

In addition to the clustering of activity in one chart, single cell variables also indicate whether or not the network formed a bump. For instance, we recorded the mean firing rate of the cells over 1 s of the network activity discarding the transient time of the activity initiation (Figure 2C). Another potential variable is the adaptation current, which contains information about the number of recent spiking events (Figure 2D). When we store four or six charts in the network, the network activity forms clear bumps in one chart and only a small number of cells were highly active. The latter point is reflected in the distribution of the mean firing rates, which peaks near zero and has a very long tail (Figure 2C, blue and green bars). When the number of stored charts was increased to eight, no bump attractor was visible and the standard deviation of the activity did not meet our clustering threshold (data not shown). In this case, the mean firing rate was distributed more evenly (Figure 2C, red bars). The difference in the distribution of the adaptation current in the two cases was much less pronounced. By contrast, the clear difference in the skewness of the mean firing rates makes it a good indicator of bump formation, which we exploit below in section “Capacity of the network.”

### 3.2 Spike-frequency-adaptation causes bump to move within and between charts

**Figure 3:**
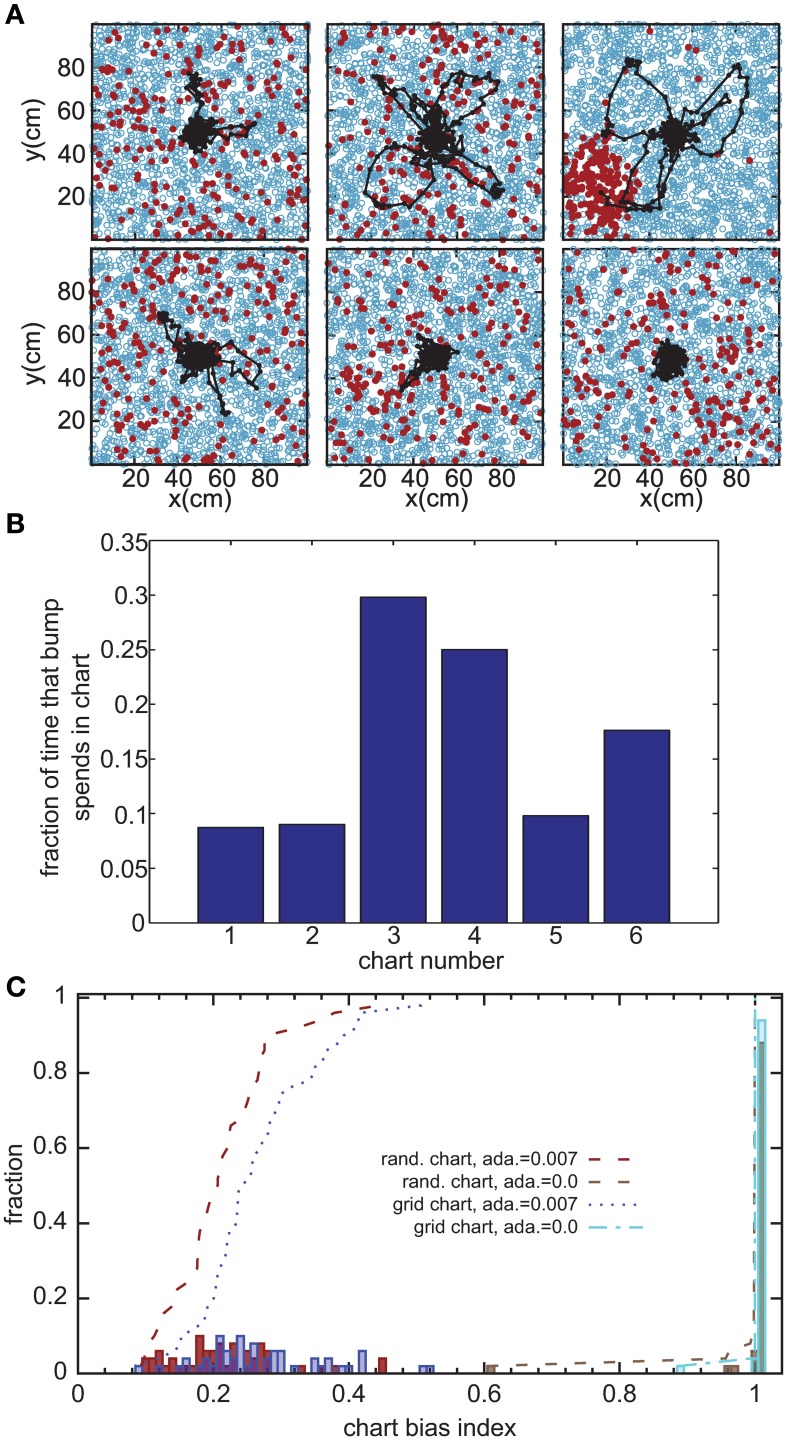
**Bump movement within and between charts induced by spike-frequency adaptation. (A)** Spike-frequency adaptation makes the bump move in the charts (displayed as in Figure 2A). Black dots represent centers of activity and the black trace shows the trajectory of bump movement. **(B)** The fraction of time that the bump stays in each chart out of the total time that the network forms a bump of activity. **(C)** Distribution of the chart bias index (see text) for different instantiations of the network. The place field centers are either scattered randomly across the environment (rand. chart) or placed in a regular grid (grid chart). The adaptation current is either disconnected from the neurons' dynamics (ada. = 0) or influencing the dynamics.

**Figure 4:**
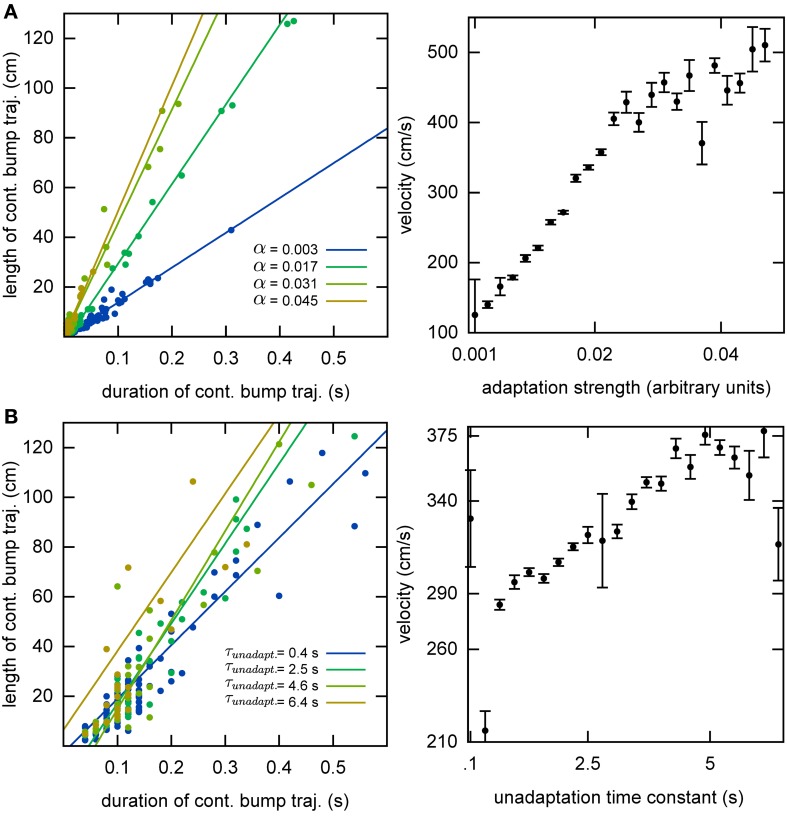
**Kinematics of the bump. (A)** The speed of the bump increases as a function of the adaptation increment. We identified continuous bump trajectories in time periods during which the standard deviation of activity was below threshold continuously in the same chart. The unadaptation time constant was τ_unadapt_ = 5 s. **(B)** The speed of the bump increases as a function of the unadaptation time constant. The adaptation increment was set at α = 0.02.

Without the adaptation current, the bump stays stationary until the end of the simulation. Even though the center of the bump jitters due to the noise in the system, the jitter amplitude is very small and hence the network cannot generate long temporal sequences across the network. That is why we need a mechanism that moves the bump of activity around. We employ an adaptation current, which models SFA. SFA moves the bump continuously in short trajectories in the neuronal sheet, making this mechanism a good candidate for a model of OSA (Figure 3A). We analyzed the kinetics of the bump movement by identifying time windows during which a stable bump remained in one chart and recorded the length of the bump's trajectory in this time window. When the trajectory lengths are plotted vs. the durations of the time windows, for a given adaptation increment α (Equation 3), the points fall on a straight line (Figure 4A). This result indicates that the bump moves predominantly at a constant speed. We further found that the mean velocity of bump propagation increases linearly with the adaptation increment for α < 0.03. For larger adaptation increments, the relationship with bump speed becomes more variable. The other parameter that governs SFA, the time constant τ_unadapt_ (Equation 2), also affects the speed of the bump movement linearly (Figure 4B). This result is in agreement with an earlier study that reported that the speed of the moving bump is modulated by the time scale of adaptation (Itskov et al., 2011a).

The adaptation current not only produces sequential activity within a chart, but also facilitates the transition between charts. It was previously conjectured that inhomogeneities in the network lead to hotspots (Renart et al., 2003; Hopfield, 2010; Itskov et al., 2011b), parts of the network in which the bump is more likely to be formed than elsewhere in the network. The stable states of a two-dimensional CANN with periodic boundary conditions, in which nodes are placed on a regular grid, are N equivalent localized bumps of activity. For large N, the energy barrier between each of these stable states vanishes and we get continuous attractors. If, however, N is small, the network is not a regular grid, or the boundary conditions are not periodic, the network forms hotspots. We investigated whether this logic applies to the movement of the bump across charts in our model. Even low levels of SFA (α = 0.007) suffice to induce bump transitions between the charts. However, the duration for which a chart hosts the activity bump is not equal for all charts (Figure 3B). To study this chart bias systematically, we defined a chart bias index as the difference between the maximum and minimum fraction of simulation time that any chart hosts the bump (Figure 3C). The smallest value of this index is zero, indicating no chart bias; the largest value is one, indicating that the bump stays in one chart for the entire duration of the simulation. The chart in which the bump of activity forms, is determined by the heterogeneities mentioned above. Confirming our qualitative observation in the preceding section in the network without adaptation current, the chart bias index is one for almost all simulation runs, indicating virtually no transitions between charts (Figure 3C, green bars). This was independent of whether the PFCs were chosen randomly or placed on a regular grid. However, when SFA was added to the neurons' dynamics, the bump spent much more even amounts of time in each chart (Figure 3C, red and blue bars) and network inhomogeneities appear to decrease, rather than increase, the chart bias. We conclude that the transition between charts driven by SFA are facilitated by network inhomogeneities because they break the symmetry between competing charts.

### 3.3 Continuous movement of the bump across a chart can account for preplay

**Figure 5:**
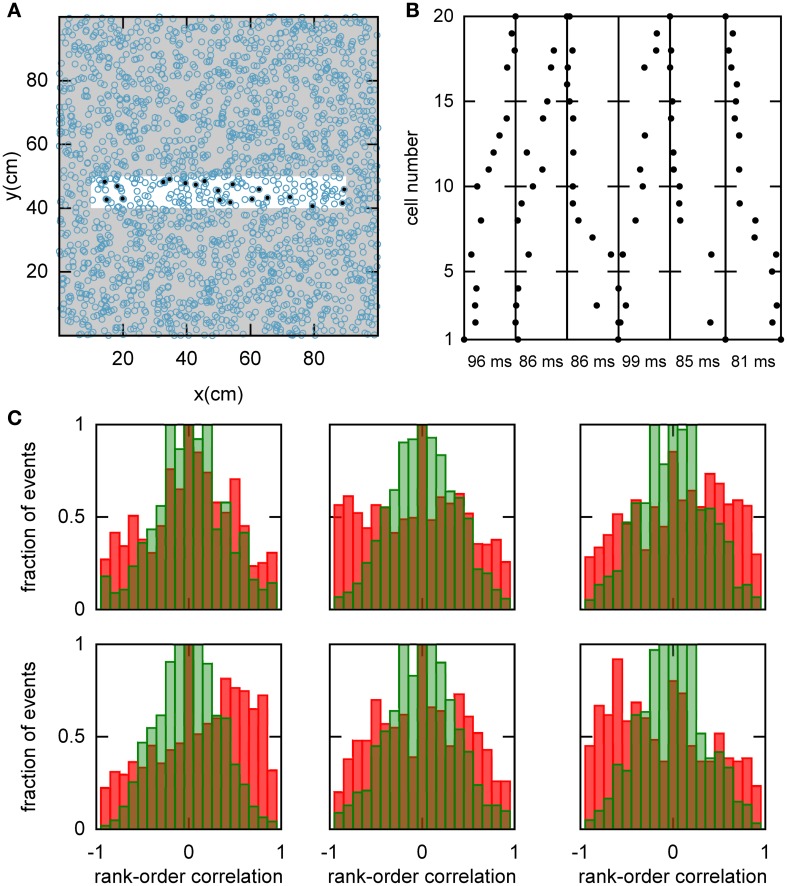
**Spatio-temporal correlations caused by continuous movement of the bump across the network in a given chart. (A)** Illustration of how a linear track (white rectangle) can be represented by a subset of neurons in a given chart (displayed as in Figure 2A). For our analysis, we randomly selected 20 neurons with place fields on the linear track to form a template (filled circles). **(B)** Spikes fired by the template neurons during selected spiking events. The place cells are sorted according to the x-coordinate of the place field centers. For this plot, we selected spiking events that show a clear positive or negative correlation between spike timing and place field orderings. **(C)** Distributions of rank-order correlations between the time of the first spike in spiking events and the spatial templates (red bars). Each of the six panels corresponds to a template drawn from one of the six charts stored in the network. Spiking events were limited to the times when the bump was located in the template's chart. Large positive and negative correlation values indicate events of forward and reverse preplay, respectively. The correlations obtained in the simulations are significantly different from the shuffled distributions (green bars; Kolmogrov–Smirnov test, *p* = 0.02, *p* < 10^−35^, *p* < 10^−20^, *p* < 10^−95^, *p* < 10^−7^, *p* < 10^−17^).

We hypothesized that preplay could be generated by the following mechanism. First, continuous movement of the bump in a chart generates OSA that is significantly correlated with the order of PFCs in that chart. Second, the network stores a small number of charts and one of the charts is reused to represent a novel environment. As a result the PFCs in the novel environment would be correlated with the OSA recorded before the first exposure to the novel environment. To examine this hypothesis, we randomly selected a set of 20 template neurons with place fields within a simulated linear track, and then identified spiking events during which a large number of the template neurons fired spikes (see Materials and Methods and Figure 5A). Selected examples of spiking events show large correlations between spike times during the spiking event and PFCs (Figure 5B). We next calculated the rank-order correlation (Equation 9) between the PFCs and those spiking events that occurred when the bump was located in the template chart (Figure 5C). A large fraction of the correlation values had large positive or negative values, suggesting that forward and reverse preplay could be found in the model. The correlations were much larger than expected by chance as indicated by a comparison of the network-generated distribution of correlations to the shuffled distribution (see Materials and Methods).

**Figure 6:**
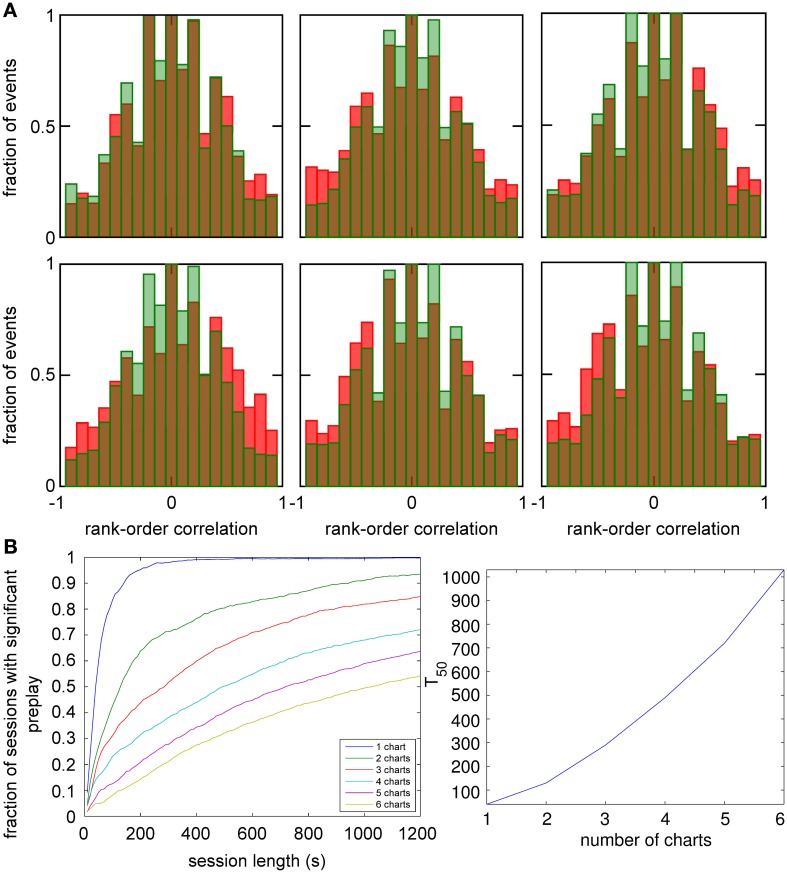
**Observing significant preplay in session data. (A)** Distributions of spatio-temporal correlations when all spiking events that involved the template neurons are included, irrespective of which chart the bump was located in during the spiking event (red bars). For each panel, the template was drawn from a different one of the six charts. The data set is the same as in Figure 5. The network-generated correlations are significantly different from the shuffled distributions (green bars; Kolmogrov–Smirnov test, *p* < 10^−5^, *p* < 10^−11^, *p* < 10^−9^, *p* < 10^−33^, *p* < 10^−13^ and *p* < 10^−21^). [**(B)**, left] Fraction of simulation runs that yield significant preplay as a function of the session length and number of charts stored in the network. [**(B)** right] The recording time required to obtain significant preplay in 50% of runs.

An analysis such as the one above is impossible in experimental data, since it is unknown which chart the bump, if it exists, is located in. The analysis has to include all spiking events that the template cells are involved in, including those when the bump is located in one of the other charts (off-chart). We therefore analyzed all spiking events generated by the network regardless of which chart the bump was in at the time of the event. The off-chart spiking events contributed mostly low correlation values (Figure 6A). This result was expected since the movement of the bump in an off-chart does not usually result in sequential activation of the neurons in the template chart. Yet, the network-generated distribution was significantly different from the shuffled distributions. This result raises the possibility that the experimentally observed preplay might be the result of the intrinsic network structure, but it is only one example.

To determine how realistic it is that an experimental recording would observe significant preplay, we repeated the simulation with many instantiations of the network and analyzed how much experimental data is required to find significant preplay. For different chart numbers, the spiking events were recorded for different session lengths. This was repeated for 100 different template selections from the same contiguous area in each chart. We then determined the fraction of simulated sessions that had significant spatio-temporal correlations (KS-test, *p* < 0.01; Figure 6B, left). For all session lengths, the fraction of sessions is lower for networks with larger number of charts. The time required to get significant preplay in 50% of the simulations increases monotonically as a function of the number of charts, in a non-linear manner (Figure 6B, right). Our results indicate that the charts in the network interfere with one another, and that this cross-talk between charts introduces spiking events with low correlations. Nevertheless, we found a high chance of observing preplay when session lengths are comparable to experimental sessions, i.e., 20 min, for up to six charts. We therefore conclude that if the number of charts stored in the network is not too high, significant preplay can result from continuous bump movement and reusing an existing chart to represent a novel environment.

Our results on the preplay of a novel linear track can also account for the preplay of the novel arm of an L-shaped track (Dragoi and Tonegawa, 2011). The two arms of the L-track could be represented either by different parts of a single chart or by two different charts. In either case, preplay of the novel arm would be equivalent to preplay of a linear track.

### 3.4 Accounting for preplay with a novel chart

**Figure 7:**
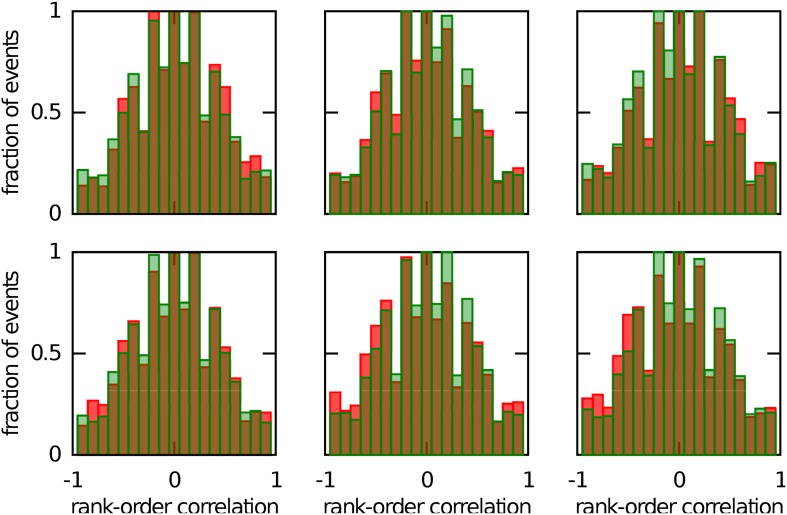
**Continuous bump movement in a given chart is not the only mechanism to get significant preplay**. When only spiking events during which the bump was located in an off-chart are included, the distributions of spatio-temporal correlations (red bars) are sometimes significantly different from the shuffled distributions (green bars; Kolmogrov–Smirnov test, *p* = 0.02, *p* = 0.02, *p* < 10^−5^, *p* = 0.42, *p* < 10^−7^ and *p* < 10^−9^). An off-chart is a chart other than the chart from which the template was drawn. The data set is the same as in Figure 5.

**Figure 8:**
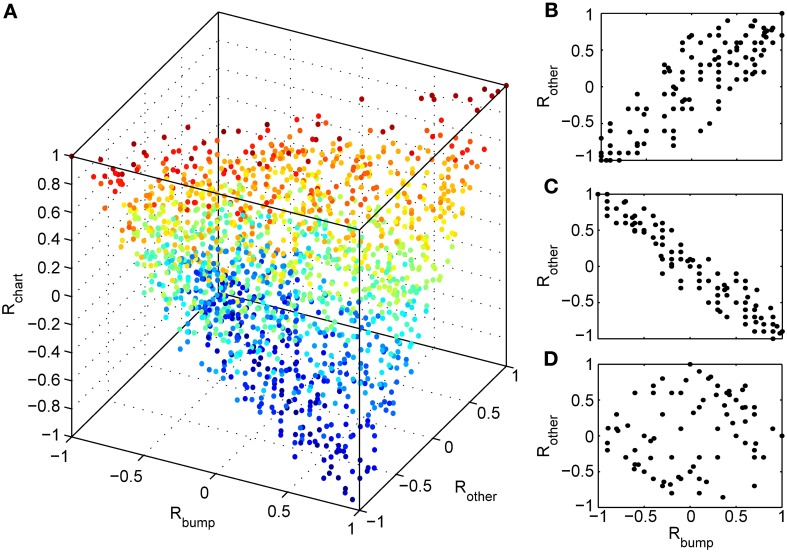
**Random correlations between charts gives rise to preplay of off-charts. (A)** Relationship between three correlation values tied to spiking events. *R*_bump_: rank order correlation between time of first spike and the positions of the corresponding cells in a template from the chart that hosts the bump; *R*_other_: rank order correlation between time of first spike and the positions of the corresponding cells in a template from an off-chart; and *R*_chart_: rank order correlation between the two templates. The color of the points change from blue (*R*_chart_ = −1) to red (*R*_chart_ = 1). The cloud of points indicates that large off-chart correlations occur only when *R*_bump_ and *R*_chart_ are large, suggesting that it is the movement of the bump and the random correlation between charts that generates significant preplay in the off-chart. **(B)** To make this even more visible we plot *R*_other_ vs. *R*_bump_ for 100 spiking events for which the corresponding *R*_chart_ is closest to 1, **(C)** for 100 events with *R*_chart_ closest to −1, and **(D)** for 100 events with *R*_chart_ values closest to zero.

We next investigated whether reusing a chart was the only way to obtain preplay in our network. We have obtained a first hint that other mechanisms are possible when we studied the correlations between PFCs and only those spiking events that occurred when the bump was located in an off-chart. As we mentioned in the preceding section, we expected these correlations to be small since the charts are generated independently of one another. This was indeed the case, but to our surprise these correlations were sometimes significant (Figure 7). To find the source of the unexpected correlations, we investigated the relationship between (A) the correlations between spiking events and the PFCs in the chart that hosts the bump *R*_bump_, (B) the correlations between spiking events and the PFCs in an off-chart *R*_other_, and (C) the correlations between the PFCs of the active cells in the template in the two charts *R*_chart_. Since we generated each chart independently of the others, PFCs in two charts quite frequently have random non-zero correlation values. Large random correlations are quite likely since they are calculated between PFCs of cells that are active in the spiking events, and by our definition, this number can be as low as five. Indeed, we found a consistent relationship between these three correlations (Figure 8). When *R*_chart_ is large and positive, *R*_bump_ and *R*_other_ are similar (Figure 8B); when *R*_chart_ is large and negative, *R*_bump_ and *R*_other_ are inversely related (Figure 8C). On the other hand, when the *R*_chart_ correlations are near zero, the relationship between *R*_bump_ and *R*_other_ vanishes (Figure 8D). We therefore conclude that correlations indicative of preplay can be introduced by random correlations between the PFCs in two charts.

**Figure 9:**
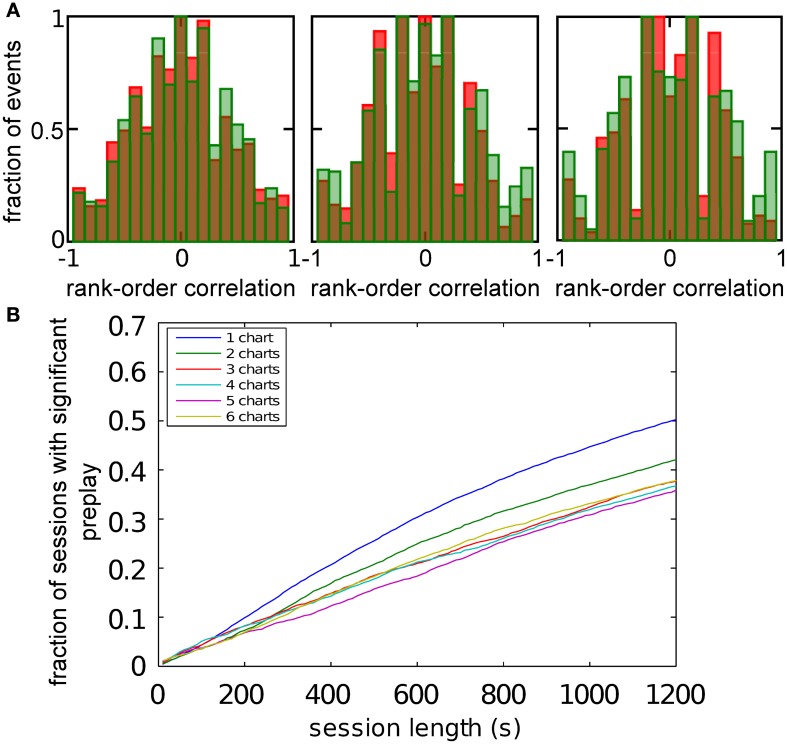
**Significant preplay of novel charts. (A)** Spatio-temporal correlations for a chart that was not present in the network when the network activity was recorded (red bars) compared to the shuffled distribution (green bars). One of the comparisons showed a significant difference (Kolomogrov–Smirnov test, *p* = 0.20, *p* = 0.01, and *p* = 0.13). **(B)** Fraction of simulation runs that show significant preplay as a function of the session length.

Continuing this line of argument, it is possible that the chart, which is preplayed, was not present in the network when the moving bump of activity generated the OSA. Any new chart, even if generated later and randomly, will have some large correlations with a pre-existing chart. We therefore simulated network activity with three charts stored in the network, then constructed a new chart, selected a template from this chart and calculated the correlations between PFCs and spiking events. Sometimes we found significant preplay, but sometimes we did not (Figure 9A). To examine this effect more closely, we repeated this analysis for 100 different selections of cells and 20 different network structures, as in Figure 6A. We found that it is possible that significant preplay of a novel chart is seen (Figure 9B), but, as expected, the likelihood is lower than that for preplay of pre-existing charts. However, the likelihoods are still high enough that the possibility cannot be excluded that preplay can be found experimentally, even when a novel environment is represented by a newly constructed chart.

### 3.5 Capacity of the network

**Figure 10:**
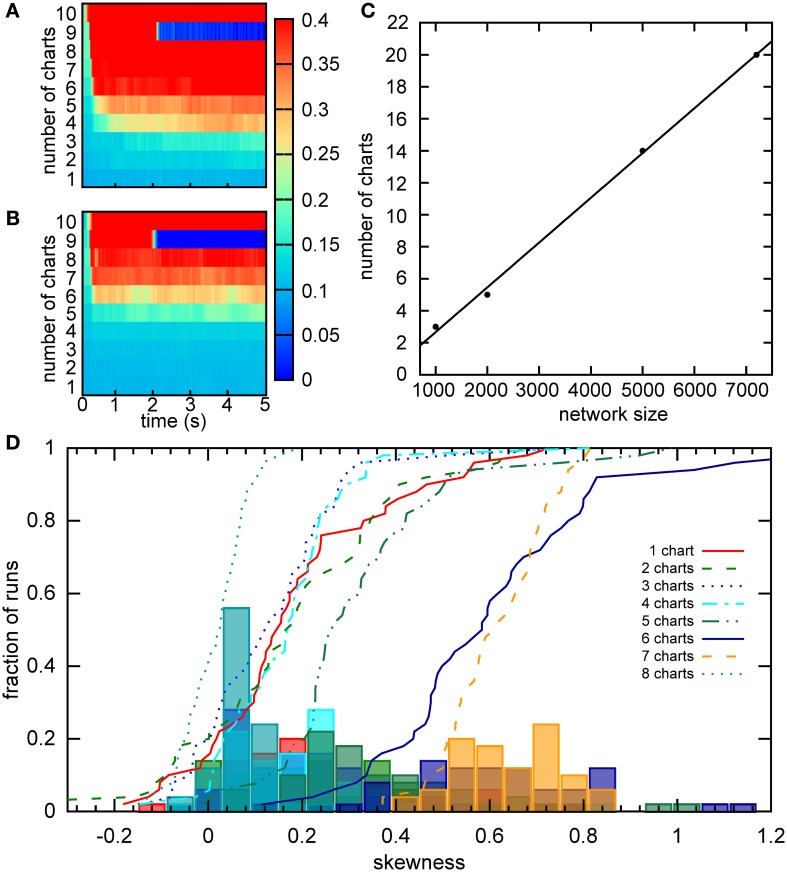
**Capacity of the network. (A)** For a given chart number the activity was initialized as a bump in each chart separately by providing bias input to nearby cells for 200 ms. The maximum of the standard deviation across charts is color coded for every time point. There is a sudden transition in this value between five to six charts, indicating a network capacity of five charts. **(B)** The same as part A, but the minimum of the standard deviation is considered. The transition occurs when the network cannot sustain the stable bump in at least one chart. Here between seven and eight charts. **(C)** The scaling of the storage capacity with the size of the network. The solid line indicates the line of best fit, 0.0028*N* − 0.14. **(D)** Distribution of the skewness of the distribution of average firing rates for networks with different numbers of stored charts. The lines represent cumulative fractions. See the text for an interpretation of these distributions.

Samsonovich and McNaughton estimated the number of stable charts in a network similar to ours to be 0.004*N*, where *N* is the number of cells in the network (Samsonovich and McNaughton, 1997). An independent, analytical estimate arrived at a similar capacity for a fully connected network (Battaglia and Treves, 1998). However, these estimates were obtained for networks without SFA and SFA can dramatically affect the activity of the network. To estimate the capacity of our network, we initiated the activity in each chart separately and observed how the activity dispersed in the chart over a 5 s period. The activity was initiated by a constant bias input *I*_bias_ = 1.92 to a clustered sub-population in a given chart. After 400 ms, the initiation input current was replaced by a constant bias current to all neurons in the network. The standard deviation of activity in the chart, in which the activity was initiated, was recorded for 5 s in 40 ms time bins. We define that a chart can support a bump if the standard deviation of the activity remains below 30 cm. Since the network can only be said to have a certain capacity, if all charts in the network can support a bump, we look for the maximum standard deviation across all charts in each time bin (Figure 10A). For our network of 2000 neurons, the capacity appears to be five (Figure 10A), which is lower than the eight predicted by Samsonovich and McNaughton's estimate, as expected. We next calculated the capacity for networks of different sizes (Figure 10C). The capacity scales linearly with the number of cells in the network 0.0028*N*, which is similar to the result reported by Samsonovich and McNaughton (1997), 0.004*N*, for a similar network without adaptation. It is surprising that the presence of adaptation that destabilizes the bump attractor only slightly reduces the coefficient without changing the qualitative relationship.

The capacity of five charts is puzzling since Figure 5 indicates that the network with six charts exhibits stable bumps in every chart. How can we reconcile the two results? One possible explanation is that the six-chart network in a given state cannot support the bump in all six charts, which was our criterion above, but changes in the network, such as the accumulation of the adaptation current, dynamically shift which charts can support the bump. Since in Figure 10A we analyzed the maximum standard deviation, we looked at the worst chart. So we would expect that some other charts are able to support the bump. To test this hypothesis, we examined the minimum standard deviation across the charts (Figure 10B). We found that even when seven charts are stored in the network, there still is at least one chart in which a bump is formed according to our criterion.

To independently verify our capacity estimate based on the activity spread, we used a measure based on a single cell property, the skewness of the distribution of mean firing rates, which was introduced in the section “Bump formation in a multi-chart continuous attractor network.” We used a short simulation time of 1 s, in which the bump does not move very far in the network, to obtain a snapshot of the network spiking activity. If we had used much longer simulations, the bump's movement would have covered the entire network and activated all cells. The distributions of skewness values across different network instantiations were relatively similar when 1–5 charts were stored in the network (Figure 10D). For six and seven charts, much larger values of skewness were encountered, suggesting a larger imbalance between the numbers of highly active and inactive cells. Then for eight charts, we see a sudden drop of the skewness toward zero indicating symmetric distributions of mean firing rates. These numbers match nicely with our analysis based on the standard deviation, where five was the maximum number of charts that could be supported by the network at any given time point, but networks with six and seven charts could still host the bump in a subset of charts. With eight charts, the network cannot support the bump at all and there is no longer an asymmetry between low and high rate neurons, i.e., no skewness of the mean firing distribution.

## 4. Discussion

We showed that a continuous attractor neural network with spike frequency adaptation that includes multiple spatial maps, or charts, can intrinsically generate sequential activity and account for preplay. Neural sequences are generated by the continuous movement of a bump of activity across the network and these temporal sequences are correlated with the order of place fields in the chart. These correlations can account for preplay in a novel environment in two different ways. Directly when an existing chart is recycled to represent the new environment, and indirectly when a new chart is generated with place fields that happen to carry some non-zero correlations with a pre-existing chart in which the bump was moving continuously.

Since our model includes excitatory recurrent connections, our neural network is a model of the CA3 area in the hippocampus. Most of the experimental results on OSA, especially the preplay sequences, were obtained from recordings in the CA1 region. Since other computational studies have shown that sequences can propagate reliably in the feedforward connections between two neural networks (Itskov et al., 2011a; Taxidis et al., 2012), we assume that the OSAs generated in CA3 are propagated to CA1.

### 4.1 Relationship to other models

The components of our model have been well studied by other authors and are fairly well understood. CANNs have been used successfully to model a number of electrophysiological observations such as phase precession (Tsodyks et al., 1996), head-direction cells (Zhang, 1996), cortical wave propagation (Pinto and Ermentrout, 2001), cortical slow wave oscillations (Ghorbani et al., 2012), and representing the spatial location of objects (Roudi and Treves, 2008). CANNs are able to store multiple memory items at the same time (Blumenfeld et al., 2006; Roudi and Treves, 2008; Romani and Tsodyks, 2010). A multi-chart structured CANN can perform path integration in the presence of idiothetic inputs (Samsonovich and McNaughton, 1997). Other studies have used CANNs to model the generation of sequential activity in the context of place cells (Hopfield, 2010; Itskov et al., 2011a).

Over the past years, many attractor models have been proposed that are capable of generating different temporal sequential activities intrinsically (Buhmann and Schulten, 1987; Dehaene et al., 1987; Herz et al., 1991). In a more recent study, Ponulak and Hopfield showed that a very similar network to that of ours with STDP learning rule can be used to mentally establish desired paths to a goal locations across multiple environments (Ponulak and Hopfield, 2013). A similar learning mechanism potentially could be used to model enhanced replay of the sequential activity in rats after an exploratory behavior. Other studies used non-attractor models accounts for sequential activity (Nadal, 1991; Leibold and Kempter, 2006; Memmesheimer, 2010; Kammerer et al., 2012). Memmesheimer proposed a random network of model neurons with supra-linear interactions that can generate propagating activity accompanying SWRs. In a recent study, Vladimirov et al. (2013) used a network of pyramidal cells with axo-axonal gap-junctions along with directional AMPA synapses to generate the replay sequences accompanying SWR oscillations. However, to our knowledge our study is the first to combine CANNs with SFA and a multi-chart structure into one model to account for preplay.

### 4.2 Electrophysiological evidence for the hypothesized mechanisms

While numerous studies have reported evidence for attractor dynamics in the hippocampus, specifically in the CA3 subregion, the existence and the type of attractor dynamics are far from certain. Some studies reported attractor dynamics for discrete states as would be expected in an autoassociative memory network (Wills et al., 2005; Jezek et al., 2011). However, another study using a very similar paradigm did not (Leutgeb et al., 2005). A recent study suggested that the difference might be an artefact of the different training protocols used in the previous studies (Colgin et al., 2010). The authors went on to propose that their results are more indicative of a continuous state attractor map, which is used for path integration (McNaughton et al., 1989; Samsonovich and McNaughton, 1997). The CANN we use in this study is based on this earlier model and shares many of its features.

Another crucial component in our network is spike frequency adaptation. Depending on the physiological source of this current, SFA can be modeled in different ways: (1) as increase in the outward synaptic conductance (Ghorbani et al., 2012), (2) as change in threshold of spiking (Itskov et al., 2011a), or (3) as an inward inhibitory current (Hopfield, 2010). We have implemented the last method in our model. In CA3, the majority of the principle cells show different levels of SFA in response to strong and moderate levels of current injections (Hemond et al., 2008).

While a moving activity bump has never been observed directly, there are some hints other than OSA, that intrinsically generated activity propagates in the hippocampal network. For instance, the position of the animal decoded from place cell spiking activity sweeps forward along the path of the animal's movement during short delays (Johnson and Redish, 2007). This observation could be interpreted in our model as follows. The bump of activity, which was initially driven by external, spatially tuned inputs, keeps moving in the absence of that input. A later study showed that the amount of this forward sweep is determined by the length of the theta cycle and modulated by the running speed of the animal (Gupta et al., 2012). It therefore appears that, in addition to adaptation, other factors such as external inputs and internal network oscillations can affect the movement of the bump in the chart. The preconfigured temporal activation of the hippocampal cells as manifested in the preplay phenomenon may function as a tool for mentally exploring future trajectories (Hopfield, 2010; Pfeiffer and Foster, 2013). The latter study reported that spatial trajectories decoded from place cell activity before goal-directed navigation in a familiar environment actually occurred during the navigation to unpredicted reward locations.

Finally, there are indications that the hippocampal network has mechanisms for predetermining which cells will be active in a novel environment in an upcoming session. Epsztein et al. observed that hippocampal CA1 cells that burst in response to depolarizing current injections in the offline state preceding exploration later exhibit place fields in a novel environment (Epsztein et al., 2011). On the other hand, cells with a more regular firing pattern remained silent in the novel environment. These observations imply that charts might be preselected and reused to represent novel environments.

### 4.3 Model predictions

A number of studies have observed intrinsically generated sequences in the hippocampus (Pastalkova et al., 2008; MacDonald et al., 2011). These time/episodic cells were shown to accurately keep track of elapsed time during the delay period in a goal directed task and predict the decision of the animal. Some time/episodic cells also have place fields outside the treadmill on which the animals are running during the delay phase (Pastalkova et al., 2008). In our reading of the data, a lot of these place fields appear to be located near the location of the treadmill. We interpret this observation to be an indirect signal of the continuous bump propagation. Due to the ongoing theta oscillation during the delay period, we believe that the network is in the online state. Although we aimed to model neural activity during the SWR state, we note that the model can also account for activity in the theta state. Online temporal sequential activity of place cells, that is modulated by the theta oscillation, have been shown to be correlated with the order of their place fields (Foster and Wilson, 2007). The timescale of these theta sequences are quite similar to OSA and it is conceivable that both theta sequences and OSA are generated by a shared network mechanism. Since the external sensory input is constantly available during the delay, we hypothesize that it can anchor the bump of activity of place cells in one position. However, the accumulated adaptation input current eventually repels the bump from its position and make it drift first to cells representing locations nearby and then to other cells representing locations outside the track. This predicts that the temporal sequences correlate with the place field ordering.

Since OSA is generated by the moving bump in our network, our model predicts that the sequences would be compressed in time if the bump moved faster. As we have shown in this paper, the bump speed depends on the adaptation increment α. This parameter, in turn, is related to the rate at which SFA accumulates. We therefore predict that if this rate were increased in CA3, e.g., by the increase in the intracellular calcium concentration, the duration of OSA would be decreased.

Our results suggest that the likelihood of observing significant preplay decreases with the number of charts stored in the network, when preplay is generated directly in the stored charts. Since reusing charts results in a higher likelihood of observing significant preplay and this likelihood decreases with the number of charts stored in the network, we therefore predict that the number of charts stored in the network is small, even though a network the size of the rat CA3 could theoretically support thousands of charts. A recent experimental study suggested that this might be the case (Dragoi and Tonegawa, 2013). This study reported that 6–7% of pre-run spiking events were distinctly correlated with place field sequences in one of three novel tracks, but not with the other two. A far smaller fraction of spiking events were significantly correlated with two or more linear tracks, indicating that each track is encoded by distinct spiking events. Extrapolating this finding to larger numbers of tracks, the authors estimated that all spiking events could distinctly code for at least 15 different tracks. While these experimental findings are highly suggestive, we are cautious to interpret them as evidence for our hypothesis that the hippocampus contains a small number of charts for two reasons. First, the fraction of preplay events that distinctly code for a single track depends on the number of cells that were recorded from but it is difficult to predict how. The number of candidate spiking events might either increase since more cells contributing spiking makes it easier to cross the threshold for the minimum number of cells firing during a spiking event, or decrease due to the spiking filling in the flanking silent periods that must surrounding the spiking event as defined by Dragoi and Tonegawa (2013). Similarly, the number of significant preplay events might either increase since sequences involving a larger number of ordered elements will lead to lower *p*-values, or decrease if the additional spikes are inconsistent with the sequential ordering of the subset of cells. The second reason for our caution is that it remains unclear how the three tracks, which were linear segments of a single U-shaped track, are represented by charts. The linear segments could be represented by different charts that are stitched together at the connection points, or they could be collectively represented by a single chart. Only in the former case would distinct codes for tracks imply the existence of distinct charts. More work, both experimental and theoretical, is needed to resolve these questions. One possible way to get at this question would be to study the chance of observing a recurrence in the spatio-temporal pattern of activity of a group of cells, such as a triplet, when recording from place cells in multiple environments. Since the recurrence probability scales with the number of stored charts, recurrences could be observable if the capacity is small enough.

Until recently, it was thought that OSA in the hippocampus could be explained solely as a replay of activity previously driven by sensory inputs. However, mounting evidence suggests a more complex scenario, in which at least some OSA such as preplay are generated by the intrinsic structure of the network. This OSA would be correlated with online activity since both are linked to the same network. Here we have suggested and studied a specific neural mechanism for this process. Since the same hippocampal network is involved in spatial memory and, arguably, in episodic memory, we speculate that similar network dynamics might generate intrinsic sequences for the storage of episodic memories (Cheng, 2013).

### Conflict of interest statement

The authors declare that the research was conducted in the absence of any commercial or financial relationships that could be construed as a potential conflict of interest.
